# Effect of Kangxianling Decoction on Expression of TGF-*β*1/Smads and Extracellular Matrix Deposition

**DOI:** 10.1155/2019/5813549

**Published:** 2019-01-01

**Authors:** Jing Ji, Liqun He

**Affiliations:** ^1^Shanghai University of Traditional Chinese Medicine, Shanghai, China; ^2^Department of Nephrology, Shuguang Hospital Affiliated to Shanghai University of Traditional Chinese Medicine, Shanghai, China

## Abstract

Kangxianling (KXL) decoction is a traditional Chinese herbal formulation which has been used to treat early and midterm chronic renal failure. Renal fibrosis is a common characteristic of progressive chronic kidney diseases (CKD). The formation of renal fibrosis is caused by kidney trauma, infection, and immune response. The pathophysiological mechanism of renal fibrosis was mainly due to increased collagen synthesis in the kidney, decreased degradation, and a large amount of extracellular matrix (ECM) deposition. The purpose of this study was intended to evaluate the effect of Kangxianling decoction on expression of TGF-*β*1/Smad signaling pathway in renal fibrosis rats. 50 specific pathogen-free Sprague Dawley (SPF SD) rats were randomly divided into five groups: control group, sham group, 5/6 nephrectomy model group, 5/6 nephrectomy model plus KXL decoction (21g /kg) group, and 5/6 nephrectomy model plus Losartan Potassium (LP) (33.3 g/kg) group. The rats were all sacrificed after two months and the left kidney tissue was sampled. HE staining was used to observe the renal pathological changes and the score of kidney damage was made. Masson staining was used to observe the degree of renal fibrosis. Immunohistochemical staining, western blot, and qRT-PCR were used to detect the expression levels of related molecules in TGF-*β*1/Smad signaling pathway. The results suggested that KXL could lighten renal histopathology damage, downregulate the expression of TGF-*β*1 (transforming growth factor-*β*1), Smad2/3, CTGF (connective tissue growth factor), Collagen I, and Collagen III, and upregulate the expression level of Smad7.

## 1. Introduction

Renal fibrosis is considered to be an irreversible process that will eventually be leading to ESRD (end-stage renal diseases). The process by which renal tubular epithelial cells lost their phenotype and transformed to mesenchymal phenotype has been shown to promote myofibroblast production and ultimately led to renal fibrosis [1–3]. It is characterized by excessive deposition of ECM, which is considered to be the pathological feature of CKD [4]. One character of renal fibrosis is the transition of tubular epithelial cells (TECs) to cells with mesenchymal characteristics, called epithelial to mesenchymal transition (EMT) [5]. Normal functioning nephrons are lost, and they are replaced by a large number of fibroblast and myofibroblast proliferation. The formation and accumulation of ECM contributed to glomerular sclerosis and renal interstitial fibrosis, eventually leading to loss of renal function. ECM is a major collagen that constitutes the structural framework of kidney tissue. So far, eighteen kinds of ECM collagens have been identified, including Collagen I, Collagen III, Collagen V, Collagen VII, Collagen IV, and Collagen FN. When EMT is activated, fibroblasts can be transformed into myofibroblasts. Myofibroblasts secrete a large number of ECM, further leading to renal fibrosis. Therefore, ECM collagens are all indicators of renal fibrosis.

TGF-*β*1/Smads signaling pathway plays a pivotal role in the thickened glomerular basement membrane and glomerulosclerosis, as well as in the occurrence and development of renal interstitial fibrosis. Intervention of this pathway has been the most intensively targets of antifibrotic therapies [6]. TGF-*β*1 is one of the key factors related to renal interstitial fibrosis. Once TGF-*β*1 is stimulated, it binds to type II TGF-*β* receptor and triggers the autophosphorylation of the type I TGF-*β* receptor, resulting in phosphorylation of Smad2 and Smad3. Later, phosphorylation of Smad2 and Smad3 bind to the common Smad4 together and form the Smad complex. The Smad complex enters the nucleus to regulate the target gene (CTGF, etc.) transcription, resulting in fibrogenesis [7]. Smad7 protein is a negative regulator of TGF-*β* signaling, which inhibits the phosphorylation of receptor-activated Smads, or binds to phosphorylated receptors competitively with Smad4, thereby preventing TGF-*β* signaling from conducting to the downstream and inhibiting Smads-mediated ECM deposition [8]. Smad2 and Smad3 are able to compete with Smad7, in order to inhibit the effect of TGF-*β*1. TGF-*β*1 has been thought to be a key mediator associated with renal fibrosis in CKD [9]. It is upregulated in both patients and animals renal injury models [10]. It was also shown that the expression of urine TGF-*β* was significantly increased in renal disease patients and animals and was positively correlated with renal fibrosis [11]. It is mainly induced by the downstream Smad signaling pathway to induce renal scarring. Once under these disease conditions, Smad2 and Smad3 are all highly activated, whereas Smad7 is degraded through the ubiquitin proteasome degradation mechanism [7]. CTGF can detect the collagen synthesis and fibrosis associated genes, which is considered to be a crucial role in the development of renal fibrosis. Inhibition of CTGF expression is a potential treatment for CKD [12, 13]. CTGF is the downstream regulatory factor of TGF-*β*1, which inhibits the transcription of Smad7, blocks the negative feedback effect on TGF-*β*1, and promotes the role of TGF-*β*1 in the process of renal fibrosis. Abnormal high-level expression of CTGF, through activating Ras/Raf/ERK signaling pathways to promote fibroblast proliferation and survival, stimulates Collagen I and Collagen III synthesis and participates in production of ECM to promote the occurrence and development of fibrotic diseases [14].

Losartan Potassium belongs to angiotensin receptor blockers (ARB) class of antihypertensive drugs. There is a large number of clinical data confirming that angiotensin converting enzyme (ACEI) inhibitors and angiotensin receptor blockers (ARB) not only can reduce the system pressure and slow down the progression of renal disease, but also can reduce the capillary pressure and the filtration protein, delay cell proliferation, and alleviate renal fibrosis which is mediated by angiotensin II [15, 16].

KXL decoction is a traditional Chinese medicine compound for strengthening Qi and removing stasis. It is composed of radix salvia miltiorrhiza (Dan Shen, from province of Shandong) 15 g, rhubarb (Zhi Da Huang, from province of Gansu) 9 g, peach kernel (Tao Ren, from province of Shandong) 12 g, radix angelicae sinensis (Dang Gui, from province of Gansu) 12 g, and radix achyranthis bidentatae (Niu Xi, from province of Henan) 15 g. All the herbal drugs were purchased from Shanghai Kangqiao Pharmaceutical Company. It is mainly used to treat renal fibrosis in the early and middle stages and has been used in the Department of Nephrology, Shuguang Hospital Affiliated to Shanghai University of Traditional Chinese Medicine for many years. In clinical practice, KXL decoction can reduce serum creatinine (Scr), blood urea nitrogen (Bun), and 24-hour urine total protein (24 h-UPro), as well as delay the development of chronic renal diseases. Kangxianling (KXL) decoction is also capable of regulating the expression of Smad2, Smad3, and Smad7 in epithelial-mesenchymal transition (EMT) [17]. Therefore, the purpose of this study is to investigate the effect of KXL on TGF-*β*1/Smads, Collagen I, and Collagen III in renal fibrosis rats and its underlying mechanism.

## 2. Materials and Methods

### 2.1. Animals and Groups

Specific pathogen-free Sprague Dawley (SPF SD) rats (200±20g) (BiKai, Shanghai, China) were used for study. The experimental animal license number was SCXK (HU) 2013-0016. The certificate number was 2008001669579 and 2008001670817. They were kept in artificial light, light and dark 12 hours each, in the experimental animal center, Shanghai University of Traditional Chinese Medicine. All animals were handled in accordance with the protocol approved by the Ethics Committee of Animal Research at the College of Shanghai University of Traditional Chinese Medicine. After one week of adaptive feeding, 50 rats were randomly divided into control group (n=10), sham group (n=10), and 5/6 nephrectomy operation group (n=30).

### 2.2. Experimental Procedure

The rats in 5/6 nephrectomy operation group were anesthetized by intraperitoneally injected with 4% sodium pentobarbital (Shanghai Pharmaceutical Factory, Shanghai, China) at a dose of 1.5 ml/kg. Their skin was shaved and the surgical area disinfected with 75% alcohol. An oblique outward incision was made starting from the left rib to remove the kidney from the retroperitoneum. After uncovering the kidney, the perirenal fat was separated from the renal capsular. Following that, two-thirds of the renal tissue was removed. Bleeding was suppressed with a gelatin sponge. When there is no longer active hemorrhage on the cut surface, the left kidney was restored and sutured the skin. After one week, the entire right kidney was removed again. Rats of sham group only made a back incision, stripped the left and right renal capsule two times, and retained adrenal gland. Then, blood was collected from the inner corner of the eye two weeks after the surgery and renal function was measured. According to no statistical difference in blood creatinine, 5/6 nephrectomy operation group rats were divided into three groups, model group, LP group, and KXL group. In two months, control group, sham group, and model group, all were given 1 ml normal saline of per 100 grams, whereas KXL group was given Kangxianling decoction by intragastric administration and LP group was given Losartan Potassium via intragastric administration. KXL decoction was made every ml containing 2.1g crude drug. The dosage translated 20 times according to the amount of clinical adult body weight. KXL group rats were all intragastric administered at a dose of 21 g/kg/d. Losartan Potassium was made into an aqueous solution of 3.33 mg of medicine per ml. The dosage also translated 20 times according to the amount of clinical adult body weight. LP group rats were all intragastric administered at a dose of 33.3 mg/kg/d.

### 2.3. Assessment of Renal Function

After two months, the rats were all sacrificed. Collect 24-hour urine of all rats with metabolism cages and determine the volume of proteinuria. The 24-hour urine samples were centrifuged at 4°C, 3000 r/min for 5 min. The blood samples, collected by abdominal aortic method, were centrifuged at 4°C, 3000 r/min for 10 min. Serum creatinine (Scr), blood urea nitrogen (Bun), and 24-hour urine protein (24 h-UPro) were all measured by automatic biochemistry instrument (Hitachi 7080, Tokyo, Japan).

### 2.4. Histological Procedure

Every renal tissue was sectioned sagittally and divided into two parts. Half of the left renal tissue was fixed in 4% paraformaldehyde; another half was snap frozen for protein and mRNA analysis. Renal samples were embedded in paraffin and cut into slices. The renal sections were stained with HE to evaluate renal structural injury, stained with Masson to assess the degree of tubular atrophy and renal fibrosis. The sections were examined by light microscopy and photographed (Zeiss Axio Lab A1). Through the tubulointerstitial lesion scoring system, HE staining scores 0-3 through interstitial dilation and inflammatory cell infiltration, with 0 indicating normal, 1 indicating mild stained (≤25%), 2 indicating moderate stained (>25%-50%), and 3 indicating intense stained (>50%). At the 200-fold field of view, five nonoverlapping tubular interstitial fields were selected and evaluated blindly for each slice, and semiquantitative analysis was performed based on the percentage of collagen area stained in the visual field. As a result of Masson staining, we used Image Pro Plus 6.0 software to determine the integrated optical density based on the same stained area in each region.

### 2.5. Immunohistochemical Staining

Paraffin-embedded kidney tissue sections 4 um were used for immunohistochemical staining analysis. They were blocked with 3% H_2_O_2_, heat-induced antigen retrieval, and washed by 1*∗*PBS for 2 times, 15 minutes each time. We add 5% blocking solution and leave the sections at room temperature for 35 minutes and then leave them to incubate overnight with Collagen I antibody (abcam, ab34710, 1:200), Collagen III antibody (abcam, ab7778, 1:800), and CTGF antibody (abcam, ab6992, 1:400) at 4°C separately. The next day, stained sections were washed by PBS for 4 times and incubated with peroxidase conjugated secondary antibodies. The tissue sections were observed with a coloring solution, washed with running water for 10 minutes, counterstained with hematoxylin for 2 minutes, rinsed for 10 minutes, dehydrated, and mounted.

### 2.6. Western Blot Analysis

Take 50 mg renal tissue from liquid nitrogen, according to the appropriate ratio, PMSF, RIPA, and Phosphatase Inhibitors were added to the centrifuge tube which contained renal samples. 14,000 r/min for 15 minutes at 4°C, BCA method to determine the protein concentration. Protein lysates were separated by 10% SDS-PAGE, then transferred to PVDF membranes (Millipore, USA), and blocked with 5% nonfat milk for one hour. The PVDF membranes were incubated overnight with primary antibodies. The primary antibodies were TGF-*β*1 (1:300; abcam, ab64715), Smad2/3 (1:1000; abcam, ab63672), Smad7 (1:3000; abcam, ab190987), CTGF (1:1000; abcam, ab6992), Collagen I (1:1000; abcam, ab34710), Collagen III (1:5000; abcam, ab7778), and GAPDH (1:5000; weiao, shanghai). The next day, membranes were washed by TBST for three times. After that, the membranes were incubated with secondary antibodies for one hour at room temperature. After another three times washed by TBST, the signal was exposed on ProteinSimple FluorChem M and used IMAGE J software to analyze the gray value.

### 2.7. Reverse Transcription-Quantitative Polymerase Chain Reaction (RT-qPCR) Detection of Gene mRNA Expression

Forward and reverse primer sequences were designed using Primer 5.0 online software (Table 1). The primer sequences were synthesized from GENIWIZ Technologies. Total RNA was extracted from renal tissue using TRIzol reagent (Invitrogen). Reverse transcription technology was used to turn RNA into cDNA. The conditions of reverse transcription reaction system were as follows: 37°C 15 min *∗*3(Reverse transcription reaction), 85°C 5 sec (Reverse transcriptase inactivation reaction), and 4°C. Gene expression levels were calculated using Roche LightCycler 480. The conditions of the amplification reaction system were as follows: predenaturation at 95°C for 30 sec, 40 cycles at 95°C for 5 sec, and 60°C for 34 sec. ΔCt = target gene Ct – GAPDH gene Ct, ΔΔCt = Ct in the experiment group - Ct in the control group. The value of Ct is the fluorescence value of amplified circulating gene. Target gene relative expression was quantified using 2-ΔΔCt method. The data was analyzed by three independent experiments and the relative mRNA expression was normalized with GAPDH as an internal reference.

### 2.8. Statistical Analysis

All values were analyzed using Spss 23.0 and the data were expressed as the mean ± standard error of the mean (SEM). Statistical analysis was using Graphpad Prism 7.0 software. Data were analyzed using one-way analysis of variance (ANOVA) to compare multiple independent sample groups, and* P*<0.05 was considered statistically significant.

## 3. Results

### 3.1. KXL Decoction Decreases the Level of Scr, Bun, and U-pro/24h in Rats

**Figure 1:**
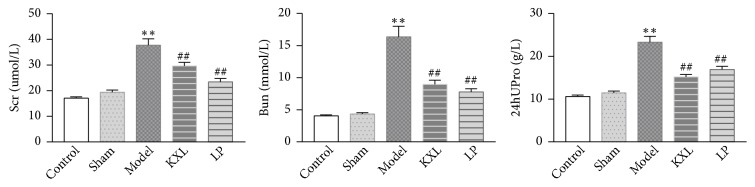
The effects of KXL and LP on the levels of Scr, Bun, and U-pro/24h in rats. Data were expressed as the mean ± standard error of mean. *∗∗P* < 0.01 compared with control group and sham group; #*P* < 0.05 and ##*P* < 0.01 compared with model group. Control, control group; Sham, sham group; Model, model group; LP, Losartan Potassium group; KXL, Kangxianling group.

After two months, there were 10, 9, 7, 8, and 7 rats in the control group, sham group, model group, KXL group, and LP group. As it is shown in Figure 1, after 5/6 nephrectomy operation, all rats were fed for two months. In the model group, Scr (serum creatinine), Bun (blood urea nitrogen), and U-pro/24 h (24 hours urinary protein) were significantly elevated compared with control group and sham group (*P* < 0.01). After two months of given KXL, the indicators above were all reduced. Scr, Bun, and U-pro/24 h were markedly restored by KXL decoction (*P* < 0.01). The result indicated that renal fibrosis model has been successfully established, and by oral administration of KXL decoction could delay renal fibrosis (Figure 1).

### 3.2. KXL Alleviates Renal Fibrosis in Rats

**Figure 2:**
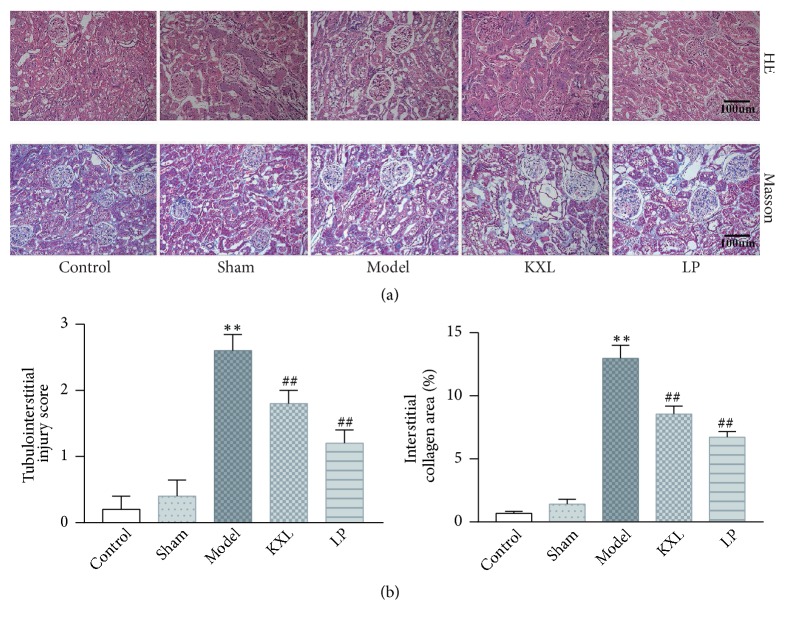
Effect of KXL and LP on renal histopathology in rats induced by 5/6 nephrectomy operation. (a) HE and Masson staining all showed renal structural damage in rats (200*∗*magnification). Masson staining illustrated the degree of renal fibrosis in rats (200*∗*magnification). (b) Both the tubulointerstitial lesion score and the interstitial collagen region were expressed as mean ± standard error of the mean. *∗∗P* < 0.01 compared with control group and sham group; #*P* < 0.05 and ##*P* < 0.01 compared with model group.

Observation with the naked eye was as follows: the residual kidney in operation group was metabolic hypertrophy. The color is dark red. There are many bulges on the surface, and there is no obvious adhesion between the surrounding tissues, so the residual kidneys are easy to separate. The glomerulus and tubular structure of the control group and the sham operation group were normal, and there was no infiltration of inflammatory cells. By contrast, HE staining showed that the residual kidney of the model group appeared with compensatory hyperplasia, mesangial matrix hyperplasia, glomerulosclerosis, glomerular necrosis and interstitial fibrosis, renal tubular interstitial tissue hyperplasia, and inflammatory cell infiltration; the area of glomerulus increased obviously. However, KXL treatment alleviated tubulointerstitial lesions compared with the model group (*P* < 0.01). Masson staining showed no abnormalities in the glomeruli of the control group and the sham group, no proliferation of mesangial cells, opening of the capillary vasospasm, and normal vascular wall interstitial. There was no obvious inflammatory cell infiltration. But in the model group, Masson staining showed glomerular hypertrophy, mesangial cells mild hyperplasia, focal segmental sclerosis, and interstitial inflammatory cell infiltration. Blue stained fibers increased significantly in the mesangial area and the glomerulus (Figure 2). However, after KXL decoction was given, glomerular lesions were reduced, mesangial cells and stromal hyperplasia were alleviated, and renal interstitial fibrosis was ameliorated (*P *< 0.01), indicating that KXL decoction could inhibit the degree of renal fibrosis in the fibrosis model induced by 5/6 nephrectomy operation.

### 3.3. KXL Slows Down Kidney Injury and Fibrosis

**Figure 3:**
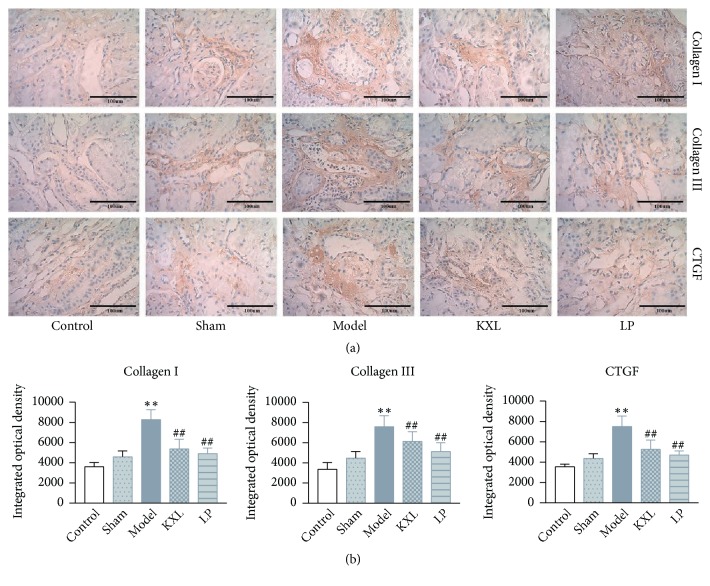
(a) Effect of KXL and LP on the expression of Collagen I, Collagen III, and CTGF in kidney samples as assessed by immunohistochemistry (400 *∗* magnification). (b) Integrated optical densities were expressed as the mean ± standard error of mean. *∗∗P* < 0.01 compared with control group and sham group; #*P* < 0.05 and ##*P* < 0.01 compared with model group.

The degree of renal fibrosis was assessed by immunohistochemistry analysis (Figure 3). The staining of Collagen I, Collagen III, and CTGF as it was shown in model rats expanded positive area compared with control group and sham group, whereas indicators above were markedly reversed after treatment with KXL (*P* < 0.01). These data demonstrated that KXL obviously reduced the overload of Collagen I, Collagen III, and CTGF on the renal remnant tissues, proving that KXL could alleviate kidney injury and fibrosis.

### 3.4. KXL Could Lighten Renal Fibrosis, Reduce the Protein Expression of TGF-*β*1, Smad2/3, CTGF, Collagen I, and Collagen III, and Increase the Protein Level of Smad7

**Figure 4:**
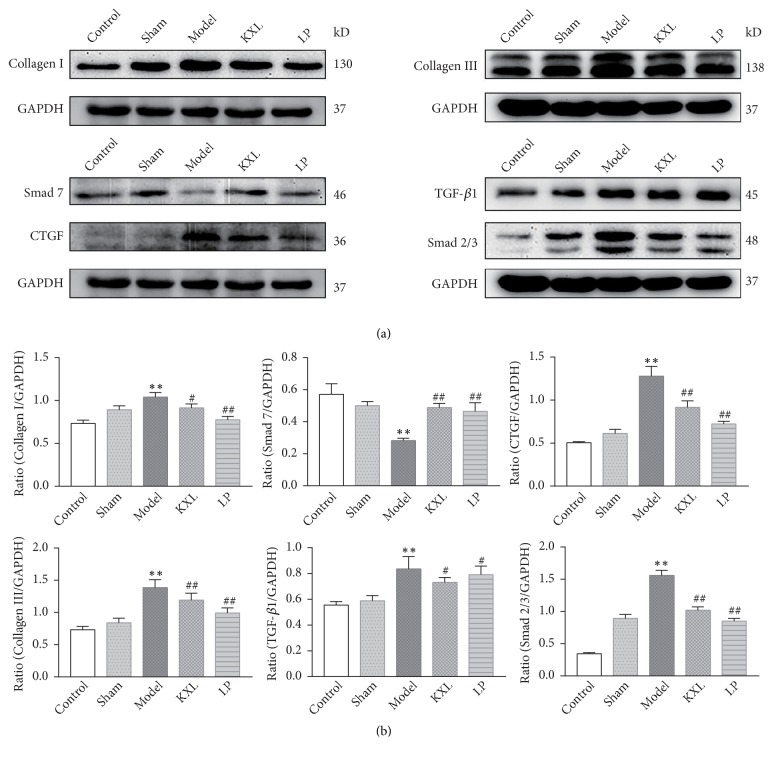
KXL alleviated 5/6 nephrectomy operation induced renal fibrosis by downregulation of TGF-*β*1, Smad2/3, CTGF, Collagen I, and Collagen III and upregulation of Smad7. (a) Representative western blot images demonstrating the expression of TGF-*β*1, Smad2/3, Smad7, CTGF, Collagen I, and Collagen III in kidney tissue from the control, sham, model, KXL, and LP groups. (b) The ratio of TGF-*β*1, Smad2/3, Smad7, CTGF, Collagen I, and Collagen III expression levels to GAPDH. Data were expressed as the mean ± standard error of mean. *∗∗P* < 0.01 compared with control group and sham group; #*P* < 0.05 and ##*P* < 0.01 compared with model group.

Compared with control group, TGF-*β*1, Smad2/3, CTGF, Collagen I, and Collagen III protein expression were increased in the model group, while the expression of Smad7 protein was decreased, the difference was statistically significant (*P *< 0.01). Compared with the model group, the expression of TGF-*β*1, Smad2/3, CTGF, Collagen I, and Collagen III protein in the LP group and KXL group was decreased, whereas the expression of Smad7 protein was increased. It was found that KXL could reduce the protein levels of TGF-*β*1, Smad2/3, CTGF, Collagen I, and Collagen III and increase the protein level of Smad7 (Figure 4). The changes were proving that KXL could alleviate renal tissue fibrosis in rats through inhibiting TGF-*β*1/Smads signaling pathway and reducing extracellular matrix accumulation.

### 3.5. KXL Ameliorates Renal Fibrosis by Downregulating the mRNA Expression of TGF-*β*1, Smad2, Smad3, Collagen I, Collagen III, and CTGF and Upregulating Smad7 mRNA Level

**Figure 5:**
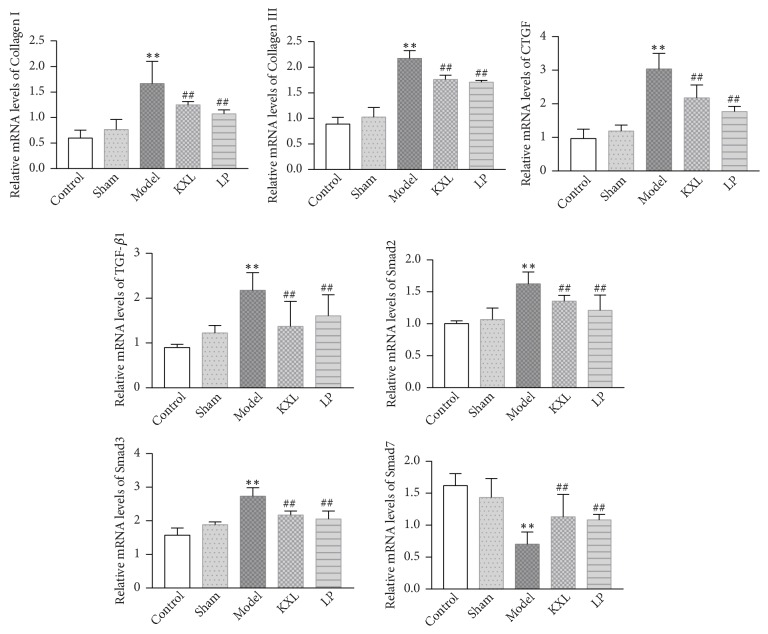
RT-qPCR analysis of TGF-*β*1, Smad2, Smad3, Smad7, CTGF, Collagen I, and Collagen III mRNA levels. Data were expressed as the mean ± standard error of mean.*∗∗P* < 0.01 compared with control group and sham group; #*P* < 0.05 and ##*P* < 0.01 compared with model group.

Compared with the control group, TGF-*β*1, Smad2, Smad3, CTGF, Collagen I, and Collagen III in the model group increased significantly, suggesting that the model was established successfully. The mRNA expression of TGF-*β*1, Smad2, Smad3, CTGF, Collagen I, and Collagen III was obviously downregulated in KXL group compared with model group, which were similar to the protein expression of them. The mRNA expression of Smad7 was markedly upregulated in KXL group, the same as Smad7 protein expression (Figure 5).

## 4. Discussion

In this article, the protective effect of KXL decoction on renal fibrosis in 5/6 nephropathy rats was studied. The pathological changes of renal fibrosis are interstitial fibroblast proliferation, fibroblast transdifferentiation into myofibroblasts, and an increase in ECM. KXL decoction is composed of radix salvia miltiorrhiza, rhubarb, peach kernel, radix angelicae sinensis, and radix achyranthis bidentatae. Modern pharmacological studies have demonstrated that each single drug in Kangxianling decoction could be useful in chronic kidney disease. For example, radix salviae miltiorrhiza was proved to be able to reverse the transdifferentiation of human renal proximal tubular epithelial cells induced by TGF-*β*1, thus delaying the procession of renal fibrosis in injured kidneys [18]. Rhubarb could alleviate tubulointerstitial fibrosis by inhibiting TGF-*β*/Smad pathway in chronic kidney disease [19]. Rhubarb also could suppress tubulointerstitial fibrosis in 5/6 nephrectomy rats by alleviating IS overload and reducing kidney oxidative stress and inflammatory injury [20]. Radix angelicae sinensis is good for preventing and treating renal tubulointerstitial fibrosis with effect similar to the angiotensin converting enzyme inhibitor (ACEI) and angiotensin receptor blocker (ARB). Its mechanism was proved to be able to improve renal fibrosis by downregulating of TGF-*β*/Smad signaling pathway and lowering the expression of angiotensin II (Ang II), a-smooth muscle actin (a-SMA), and fibronectin (FN) [21]. Radix achyranthis bidentate could slow down the progression of renal fibrosis by inhibiting the activation TGF-*β*/Smad pathway and snail expression [22]. Peach kernel is a traditional Chinese medicine for promoting blood circulation and removing blood stasis. It could protect renal tubular cells and slow down renal interstitial fibrosis by downregulating the expression of CTGF [23]. KXL decoction has been used in the treatment of nephrotic syndrome, hematomas, and chronic glomerulonephritis. It has noticeable effects on removing stasis and activating blood, in order to restore renal function. Previous studies have shown that KXL decoction could significantly improve renal function in 5/6 nephrectomy rats by inhibiting P38MARK signaling pathways and reducing the expression of NF-kb, *α*-SMA, TNF-*α*, and IL-6 [24, 25]. The study showed that KXL treatment is a classic prescription for Chinese medicine and has a protective effect on renal fibrosis.

KXL decoction significantly decreased the level of Scr, Bun, and U-pro/24 h in rats. After 5/6 nephrectomy operation, Scr and Bun were all elevated because the kidneys could not completely excrete metabolites and harmful substances in the body. U-pro/24 h elevated also reflected kidney damage. Scr, Bun, and U-pro/24 h in KXL group were obviously restored, indicating that KXL decoction could improve renal function. HE and Masson staining showed glomerular sclerosis, renal interstitial fibrosis, and inflammatory cell infiltration. These pathology changes were all alleviated after treatment with KXL decoction. The results suggested that KXL decoction could alleviate renal pathological changes in 5/6 renal ablation rats.

TGF-*β*1 has been recognized as a key mediator in the pathogenesis of progressive renal fibrosis [26, 27]. It is an active polypeptide that is closely related to various tissues and organs. The fibrosis promotion effect of TGF-*β*1 was transmitted through the downstream Smads family. TGF-*β*1 plays an important role in tubulointerstitial fibrosis; biological effects are mediated by activated signal transduction pathways, whereas Smads proteins are important for maintaining the TGF-*β*1 signal transduction pathway. TGF-*β*1 is also a key regulator of ECM; TGF-*β*1/Smads signal transduction is involved in the synthesis and degradation of ECM (including Collagen I and Collagen III). TGF-*β*1 could also be served as a biomarker for renal fibrosis, since increased level of TGF-*β*1 has been detected in renal diseases [28–30].

Smad is considered to be the most classic pathway for TGF-*β*1 induced renal fibrosis [31]. Smad2/3 is a key downstream mediator responsible for the biological effects of TGF-*β*1. It is strongly activated in kidney disease [32]. Smad7 has been identified as a negative regulator of TGF-*β*1/Smads signaling. It is one type of inhibitory Smad protein. It can regulate Smad2/3 activation through its negative feedback mechanism. Once Smad7 is degraded through the ubiquitin proteasome degradation mechanism, Smad2/3 is activated, and renal fibrosis is enhanced [33, 34]. For the entire Smads signaling pathway, the activation of Smad2/3 enhances the effect of TGF-*β*1 signaling, thereby promoting fibrosis formation. In contrast, the activation of Smad7 inhibits the biological effects of the TGF-*β*1/Smads signaling pathway, thereby preventing the formation of fibrosis.

The progression of CKD is characterized by progressive fibrosis of the kidney. This fibrosis reflects an increase in collagen deposition, such as Collagen I and Collagen III. Collagen I and Collagen III are the main components of the ECM and are thought to play a major role in renal interstitial fibrosis. CTGF is a downstream regulatory factor of TGF-*β*1 and promotes the role of TGF-*β*1 in the process of renal fibrosis. CTGF is involved in initiating and triggering the development of renal injury to promote fibrosis; inhibition of CTGF expression has been identified as a potential target for the treatment of CKD. CTGF may also contribute to the accumulation of mesangial matrix in chronic glomerular disease, leading to the development of glomerular sclerosis [35].

Therefore, in this article, we could know that KXL decoction played an important role in alleviating renal fibrosis by inhibiting the expression of TGF-*β*1, Smad2, Smad3, Collagen I, Collagen III, and CTGF and promoting the expression of Smad7, thereby reducing the occurrence and development of renal fibrosis.

In this study, we observed that KXL decoction could improve renal fibrosis in 5/6 nephrectomy model. KXL decoction could downregulate the TGF-*β*1/Smads signaling pathway and reduce ECM accumulation, thereby delaying the progression of renal fibrosis.

## 5. Conclusion

This study revealed that KXL decoction could alleviate renal fibrosis by inhibiting TGF-*β*1/Smads signaling pathway and reducing extracellular matrix deposition.

## Figures and Tables

**Table 1:** List of primer sequences for reverse transcription-quantitative polymerase chain reaction.

Gene	Forward primer	Reverse primer
TGF-*β*1	TGCGCCTGCAGAGATTCAAG	AGACAGCCACTCAGGCGTAT
Smad2	GCAGGTGGTGGGAACAGAAT	CCGTATTTGCTGTACTCAGTCCC
Smad3	CAGGAGGAGAAGTGGTGCGA	TGGTGTTCACGTTCTGCGTG
Smad7	AAGTGTTCAGGTGGCCGGAT	TGGACAGTCTGCAGTTGGTT
CTGF	TCCGGACGCCTAAAATTGCC	CCATCGGGGCACTTGAACTC
Collagen I	TGACGCATGGCCAAGAAGAC	CCGTGCCATTGTGGCAGATA
Collagen III	GGACAGATGCTGGTGCTGAAG	GGACAGATGCTGGTGCTGAAG
GAPDH	GGCACAGTCAAGGCTGAATG	ATGGTGGTGAAGACGCCAGTA

## Data Availability

The data used to support the findings of this study are available from the corresponding author upon request.
